# Ultrasound Echotexture Analysis as a Diagnostic Adjunct for *Naja atra* Envenomation Versus Cellulitis in Acute Limb Swelling

**DOI:** 10.3390/diagnostics16213594

**Published:** 2026-10-30

**Authors:** Ting-Kuang Yeh, Yan-Chiao Mao, Hsien-Po Huang, Po-Hsiu Huang, Chien-Hao Tseng, Chih-Sheng Lai, Po-Yu Liu, Kuo-Lung Lai

**Affiliations:** 1 Division of Infectious Diseases, Department of Internal Medicine, Taichung Veterans General Hospital, Taichung 407219, Taiwan; 2 Institute of Clinical Medicine, National Yang Ming Chiao Tung University, Taipei 112304, Taiwan; 3 Department of Post-Baccalaureate Medicine, College of Medicine, National Chung Hsing University, Taichung 402202, Taiwan; 4 PhD Program in Medical Biotechnology, College of Medical Science and Technology, Taipei Medical University, Taipei 110301, Taiwan; 5 Department of Medical Toxicology, Taichung Veterans General Hospital, Taichung 407219, Taiwan; 6 College of Medicine, National Defense Medical University, Taipei 114201, Taiwan; 7 School of Medicine, College of Medicine, National Yang Ming Chiao Tung University, Taipei 112304, Taiwan; 8 Institute of Molecular Biology, National Chung Hsing University, Taichung 402202, Taiwan; 9 Division of Plastic and Reconstructive Surgery, Department of Surgery, Taichung Veterans General Hospital, Taichung 407219, Taiwan; 10 Doctoral Program in Translational Medicine, Rong Hsing Translational Medicine Research Center, National Chung Hsing University, Taichung 402202, Taiwan; 11 Infection Control Center, Taichung Veterans General Hospital, Taichung 407219, Taiwan; 12 Division of Allergy, Immunology and Rheumatology, Department of Internal Medicine, Taichung Veterans General Hospital, Taichung 407219, Taiwan

**Keywords:** *Naja atra* envenomation, cellulitis, echotexture analysis, gray-level co-occurrence matrix, ultrasound

## Abstract

**Objectives:** *Naja atra* (*N. atra*) envenomation and cellulitis frequently present with overlapping clinical features of acutely swollen extremities, creating a diagnostic dilemma during the early clinical course of acute limb swelling. This study investigated whether ultrasound imaging with echotexture analysis can effectively differentiate these two conditions. **Methods:** This retrospective cross-sectional study conducted at Taichung Veterans General Hospital included 115 ultrasound images from 84 patients categorized into three groups: confirmed *N. atra* envenomation (*n* = 45), confirmed non-snakebite cellulitis (*n* = 40), and controls (*n* = 30). Gray-level co-occurrence matrix (GLCM) features including contrast, correlation, energy, and homogeneity, along with the grayscale value and the power Doppler vascularity-to-ROI ratio, were quantitatively computed from ultrasound images. Group comparisons used generalized estimating equations to account for multiple images from the same patient, with Bonferroni correction for pairwise comparisons. Diagnostic accuracy was evaluated using receiver operating characteristic (ROC) curve analysis. **Results:** Significant differences among the three groups were found for contrast (*p* < 0.001), correlation (*p* < 0.001), homogeneity (*p* = 0.005), and the vascularity-to-ROI ratio (*p* < 0.001). In pairwise comparisons between *N. atra* envenomation and cellulitis, contrast was significantly higher (0.273 ± 0.068 versus 0.230 ± 0.040, *p* = 0.001) and homogeneity was lower (0.878 ± 0.020 versus 0.890 ± 0.017, *p* = 0.004) in the *N. atra* group. ROC curve analysis for discriminating *N. atra* envenomation from cellulitis yielded an Area Under the Curve of 0.707 (95% confidence interval, 0.591–0.824) for contrast and 0.684 (95% confidence interval, 0.568–0.800) for homogeneity. At an optimal cutoff of ≥0.243, contrast achieved 73.3% sensitivity and 70.0% specificity. **Conclusions:** Echotexture analysis, specifically contrast and homogeneity, may differentiate *N. atra* envenomation from cellulitis. These parameters may reflect the heterogeneous tissue destruction induced by venom compared to the diffuse inflammation seen in cellulitis. These proof-of-concept findings require validation in larger prospective cohorts before clinical application.

## 1. Introduction

*Naja atra* (*N. atra*) envenomation causes severe local tissue inflammation that frequently presents with clinical features like those of common bacterial soft tissue infections. Both conditions are characterized by localized swelling, warmth, and erythema, which makes accurate clinical differentiation difficult in the initial evaluation, especially when information about the snakebite is unavailable. *N. atra* envenomation necessitates prompt administration of specific antivenoms, while cellulitis is managed with antibiotic therapy and supportive care. An accurate and timely diagnosis is critical because delayed treatment of venomous injury increases the risk of extensive tissue necrosis and systemic complications, whereas unnecessary antivenom administration can lead to potential allergic reactions and the depletion of limited medical resources [1,2].

In Taiwan, *N. atra* is responsible for a significant proportion of medically important snakebites. *N. atra* envenomation frequently leads to secondary cellulitis because venom-induced tissue necrosis provides a substrate for bacterial invasion [3]. Clinical differentiation is often difficult because patients with *N. atra* bites who develop superimposed cellulitis exhibit physical findings that are nearly identical to those with primary bacterial cellulitis. Furthermore, laboratory markers including leukocyte count, C-reactive protein, and creatine kinase lack the necessary sensitivity and specificity for definitive diagnosis in the acute setting, and specialized venom detection assays are not widely available.

Point-of-care ultrasound (POCUS) provides a real-time imaging modality that may assist in resolving this diagnostic ambiguity. Conventional B-mode ultrasound features of soft tissue infections such as the cobblestone pattern from interstitial edema, fascial thickening, and subcutaneous fluid collections have been previously described [4,5,6]. Recent studies have demonstrated the utility of ultrasound in characterizing venom-induced tissue inflammation and assessing the risk of compartment syndrome in snakebite patients [7,8]. However, these qualitative characteristics are operator-dependent and often lack specificity for differentiating between venom-induced tissue destruction and bacterial cellulitis. A quantitative approach to image analysis could potentially offer the objectivity required for reliable clinical differentiation.

Echotexture analysis using the gray-level co-occurrence matrix (GLCM) is a computational method that extracts statistical features based on the spatial relationships between pixel intensities [9]. These features, which include contrast, correlation, energy, and homogeneity, characterize tissue microarchitecture quantitatively and reduce reliance on subjective visual interpretation. This technique has shown utility in musculoskeletal imaging, notably in a study by Lai et al. that identified structural changes in the supraspinous enthesis that were not detectable by conventional visual inspection [10].

The greatest diagnostic uncertainty arises when the history of snake exposure is unreliable, such as after an unwitnessed bite. A quantitative imaging marker that distinguishes venom-induced tissue change from bacterial cellulitis could serve as a useful adjunct in this scenario. Currently, no studies have applied quantitative echotexture analysis to the differentiation of *N. atra* envenomation and cellulitis. This study hypothesized that venom-induced tissue destruction and bacterial soft tissue infection produce distinct ultrasound texture signatures because they result from different pathophysiological processes, specifically heterogeneous tissue necrosis and hemorrhage compared with diffuse interstitial edema. The primary objective of this study was to compare GLCM features and power Doppler vascularity among *N. atra* envenomation, cellulitis, and control groups while also evaluating the diagnostic accuracy of these features for discriminating *N. atra* envenomation from cellulitis.

## 2. Materials and Methods

### 2.1. Study Design and Setting

This retrospective cross-sectional study was conducted at Taichung Veterans General Hospital (TCVGH), a tertiary medical center in central Taiwan, utilizing ultrasound data collected between November 2013 and February 2026. The study protocol was approved by the Institutional Review Board of TCVGH (IRB No. CE26160A). Due to the retrospective nature of the analysis, the requirement for informed consent was waived.

### 2.2. Study Population and Group Definitions

The study population comprised three distinct groups: *N. atra* envenomation, non-snakebite cellulitis, and a control group. To ensure diagnostic accuracy, all medical records and clinical diagnoses for the envenomation and cellulitis groups were independently reviewed by two infectious disease specialists, with reference to the clinical symptoms and signs documented in the medical records. Only one species in the genus *Naja*, *N. atra*, is distributed in Taiwan. This study included only patients bitten by *N. atra*. The species was identified based on examination of snake specimens or photographs. The *N. atra* envenomation group included patients presenting with acute limb swelling following these confirmed bites, alongside fang marks or a clinical course typical of *N. atra* envenomation as recorded in their clinical charts. The cellulitis group consisted of patients with non-snakebite cellulitis diagnosed by acute-onset erythema, warmth, and tenderness as noted in the medical documentation, covering various etiologies including simple cellulitis, abscess formation, and infections associated with diabetes mellitus. The control group included patients who underwent musculoskeletal ultrasound for musculoskeletal symptoms and had preserved skin structure at the examined anatomical sites.

### 2.3. Quantitative Data Acquisition and Analysis

All ultrasound examinations were performed by a single experienced rheumatologist (KL Lai) with 15 years of musculoskeletal ultrasound experience, certification by the European Alliance of Associations for Rheumatology (EULAR), and a EULAR competency level of 1 for musculoskeletal ultrasound, ensuring standardized acquisition and image quality across the entire study period. All examinations were performed using the same ultrasound system (Philips iU22; Philips Ultrasound, Inc., Bothell, WA, USA) equipped with a 5–12 MHz linear transducer and power Doppler function, with consistent settings applied throughout. For power Doppler, the pulse repetition frequency was set at 500 Hz, and the color gain was adjusted until no power Doppler signal was detected below the bone cortex; the room temperature was maintained at 25 °C during all examinations. In cases where multiple ultrasound scans were performed on the same patient (e.g., at different anatomical locations or different time points during the clinical course), all images were included, and the correlation between images from the same patient was accounted for in the statistical analysis.

**Figure 1:**
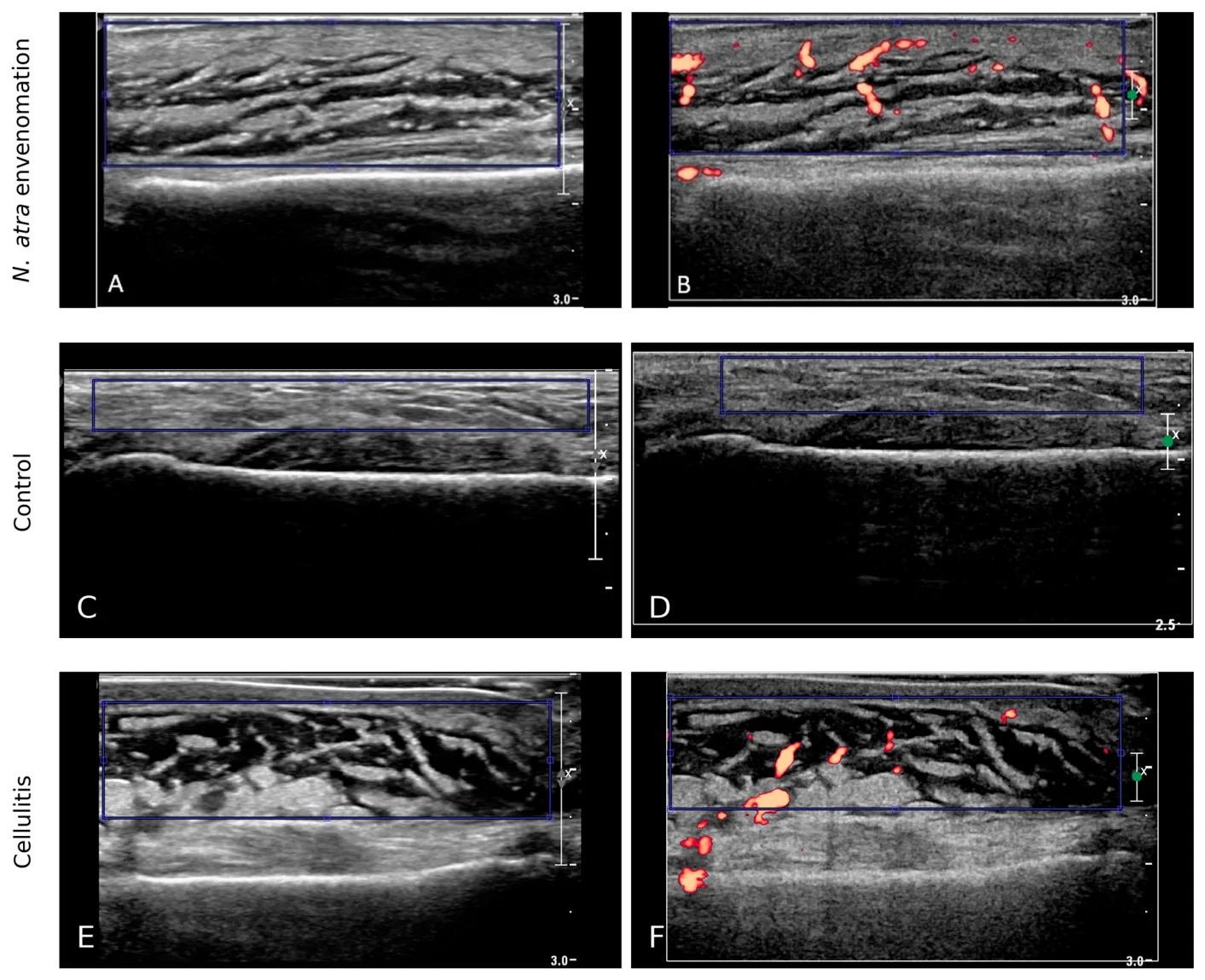
Representative grayscale (left) and power Doppler (right) ultrasound images of the subcutaneous tissue in each group. (**A**,**B**) *N. atra* envenomation at the right dorsal hand in a man. (**C**,**D**) Control. (**E**,**F**) Cellulitis. The blue box indicates the region of interest (ROI), which covers most of the subcutaneous tissue at the examined site. Color pixels within the ROI on the power Doppler images were used to calculate the vascularity-to-ROI ratio.

The ultrasound images were analyzed using a well-documented echotexture approach [10,11]. A representative region of interest (ROI) of the subcutaneous tissue was determined on each image by the same rheumatologist who performed the examinations. ROI delineation was performed retrospectively on de-identified images, generally more than six months after image acquisition. The ROI was drawn to cover most of the affected subcutaneous tissue and avoided extra-subcutaneous tissues (Figure 1). Six specific parameters were extracted to characterize the soft tissue, beginning with the grayscale (GS) value (range 0–255), which represented the mean of pixel intensities to reflect overall tissue echogenicity. Second, texture features were further computed using the GLCM algorithm with an angular displacement of 0 degrees. These features included Contrast to measure intensity differences between neighboring pixels, Correlation to quantify the linear dependency of pixel patterns, Energy to reflect the sum of squared matrix elements for texture uniformity, and Homogeneity to measure the closeness of the distribution to the diagonal as an indicator of image smoothness. Finally, power Doppler vascularity was quantified as the vascularity-to-ROI ratio, defined as the percentage of color pixels within the ROI (the number of color pixels divided by the total number of pixels in the ROI) to provide an objective measurement of tissue hyperemia.

### 2.4. Statistical Analysis

Continuous variables were expressed as mean ± standard deviation and median (interquartile range [IQR]). Patient characteristics were compared using the Mann–Whitney U test for continuous variables and the chi-square test for categorical variables. Because some patients contributed more than one image, ultrasound features were compared using generalized estimating equations (GEE) with an exchangeable working correlation structure and robust standard errors, with the patient as the clustering unit. The vascularity-to-ROI ratio was log-transformed (log[x + 1]) before modeling. The overall difference among the three groups was assessed using a Wald test, followed by pairwise comparisons, for which unadjusted *p* values are reported. The Bonferroni-adjusted significance threshold for pairwise comparisons was *p* < 0.017.

The diagnostic performance of GS value, GLCM texture features and the vascularity-to-ROI ratio in discriminating *N. atra* envenomation from cellulitis was evaluated using Receiver Operating Characteristic (ROC) curve analysis. The Area Under the Curve (AUC) was calculated along with its 95% confidence interval (CI), which was estimated using the nonparametric method for clustered ROC data described by Obuchowski to account for multiple images from the same patient [12]. Optimal cutoff values were determined at the image level using the Youden index, from which accuracy, sensitivity, specificity, positive predictive value, and negative predictive value were derived. A patient-level sensitivity analysis was performed using only the first ultrasound image from each patient, in which the *N. atra* envenomation and cellulitis groups were compared using the Mann–Whitney U test with unadjusted *p* values, and the 95% CIs of the AUC were estimated using the DeLong method. To examine the influence of examination timing, the association between the time from emergency department presentation to the first ultrasound examination and each texture feature was assessed in the *N. atra* envenomation group using Spearman correlation. In addition, the first images were stratified at 72 h after presentation and compared with the cellulitis group using the Mann–Whitney U test. The patient examined at an outpatient visit after discharge was excluded from this time-stratified analysis. All statistical analyses were two-tailed, with *p* < 0.05 considered statistically significant for omnibus tests and Bonferroni-adjusted thresholds applied for post hoc pairwise comparisons. Generative AI tools (Claude Sonnet 4.6 and Claude Opus 5.5, Anthropic, San Francisco, CA, USA) were used to assist in generating the Python (version 3.13) code for the statistical analyses and figures. All figures were generated from the study data using Python on the Google Colab platform, and the authors reviewed and verified all outputs.

## 3. Results

### 3.1. Study Population

A total of 115 ultrasound images were included in the final analysis, obtained from 84 individual subjects (Supplementary Figure S1). The study population consisted of three groups: the *N. atra* envenomation group (*n* = 45 images) from 27 patients (17 males [63.0%] and 10 females [37.0%], age 55.9 ± 16.5 years), the cellulitis group (*n* = 40 images) from 40 patients (24 males [60.0%] and 16 females [40.0%], age 61.7 ± 18.1 years), and the control group (*n* = 30 images) from 17 patients (6 males [35.3%] and 11 females [64.7%], age 51.5 ± 20.2 years). All *N. atra* envenomation cases received antivenom treatment, and all cellulitis cases were managed with antibiotic therapy without antivenom administration. Accordingly, the two disease groups were intended to represent the clinical entities that the ultrasound analysis aimed to distinguish. These were non-snakebite cellulitis on the one hand, and *N. atra* envenomation irrespective of any superimposed secondary cellulitis on the other.

In the *N. atra* envenomation group, the ultrasound images targeted the hand (*n* = 17) and foot (*n* = 28). For the cellulitis group, affected sites included the hand/forearm (*n* = 16) and foot/leg (*n* = 24). The distribution of anatomical sites did not differ significantly between the two disease groups (*p* = 0.83). The control group underwent ultrasound imaging of the hand and foot as baseline comparisons. In the *N. atra* envenomation group, the median time from emergency department presentation to the initial ultrasound examination was 30.5 h (IQR, 16.3–66.4; range, 2.6–449.2 h). Examinations obtained more than 72 h after presentation were performed during hospitalization, except for one patient who was examined at an outpatient follow-up visit after discharge (449.2 h). The demographic and clinical characteristics of the two disease groups are summarized in Table 1. The *N. atra* envenomation and cellulitis groups were comparable in sex distribution and age. White blood cell count, neutrophil percentage, and lymphocyte percentage did not differ significantly between the groups. C-reactive protein was significantly higher in the cellulitis group than in the envenomation group (median 1.42 versus 0.15 mg/dL, *p* = 0.009).

### 3.2. Three-Group Comparison of Echotexture Features

After accounting for multiple images from the same patient, significant differences among the three groups were observed for Contrast (*p* < 0.001), Correlation (*p* < 0.001), Homogeneity (*p* = 0.005), and the vascularity-to-ROI ratio (*p* < 0.001), but not for GS value (*p* = 0.139) or Energy (*p* = 0.247). The descriptive statistics for these parameters are detailed in Table 2.

Pairwise post hoc comparisons (with a Bonferroni-adjusted significance threshold of *p* < 0.017) identified specific differences between groups. In the comparison between *N. atra* envenomation and cellulitis, the *N. atra* group exhibited significantly higher Contrast (0.273 ± 0.068 vs. 0.230 ± 0.040, *p* = 0.001) and lower Homogeneity (0.878 ± 0.020 vs. 0.890 ± 0.017, *p* = 0.004). No significant difference was observed between these two groups in GS value (*p* = 0.226). The vascularity-to-ROI ratio was lower in the *N. atra* group than in the cellulitis group (median 2.08% vs. 3.09%, *p* = 0.037), but this difference did not reach the Bonferroni-adjusted threshold.

**Figure 2:**
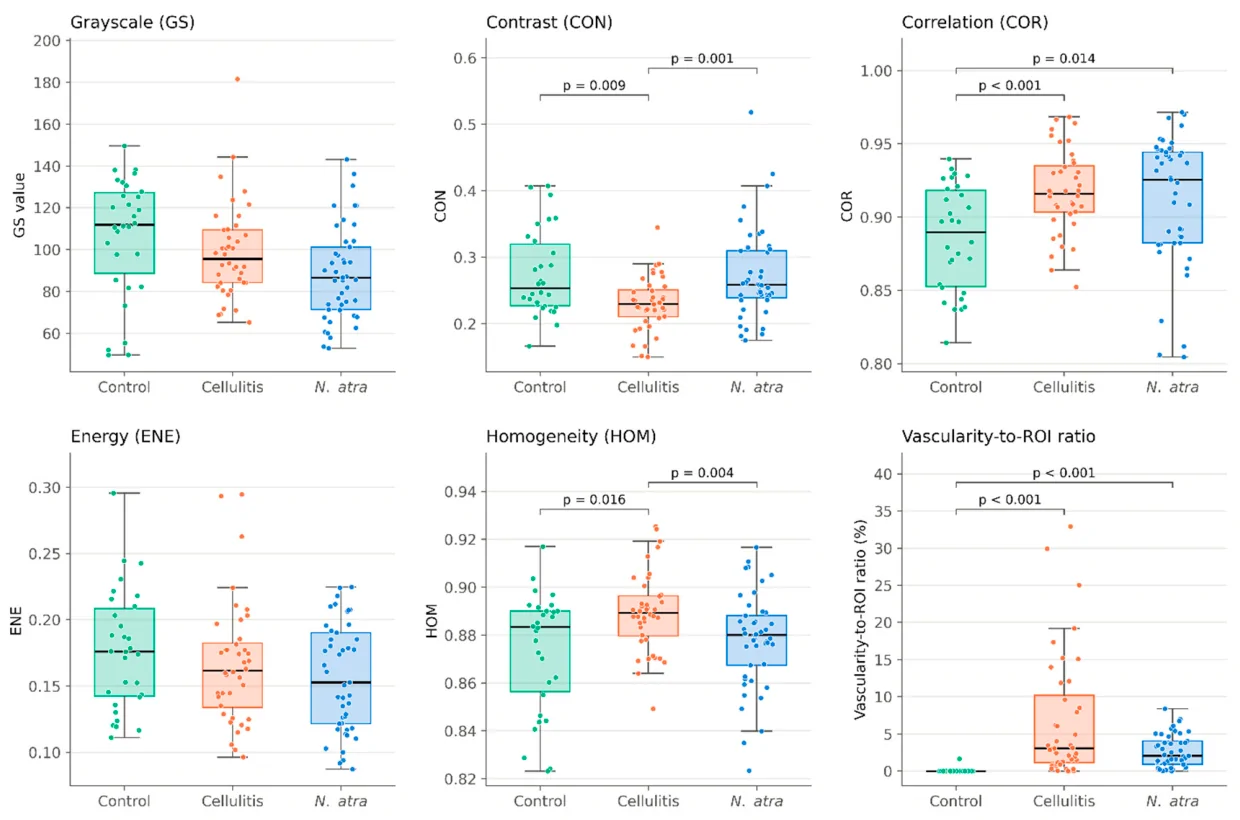
Boxplots of ultrasound features across the three groups. Each dot represents one image. Boxes indicate the median and interquartile range, and whiskers extend to 1.5 times the interquartile range. *p* values were derived from generalized estimating equations accounting for multiple images from the same patient, and only pairwise comparisons meeting the Bonferroni-adjusted threshold (*p* < 0.017) are shown. The vascularity-to-ROI ratio is expressed as the percentage of color pixels within the ROI. GS, grayscale. CON, contrast. COR, correlation. ENE, energy. HOM, homogeneity. ROI, region of interest.

Regarding comparisons with the control group, the cellulitis group showed significantly lower Contrast (*p* = 0.009), higher Correlation (*p* < 0.001), and higher Homogeneity (*p* = 0.016) than the control group, and the *N. atra* group showed higher Correlation than the control group (*p* = 0.014). Both the *N. atra* and cellulitis groups demonstrated significantly higher vascularity-to-ROI ratios compared with the control group (both *p* < 0.001) (Figure 2).

### 3.3. Diagnostic Accuracy for N. atra Envenomation Versus Cellulitis

**Figure 3:**
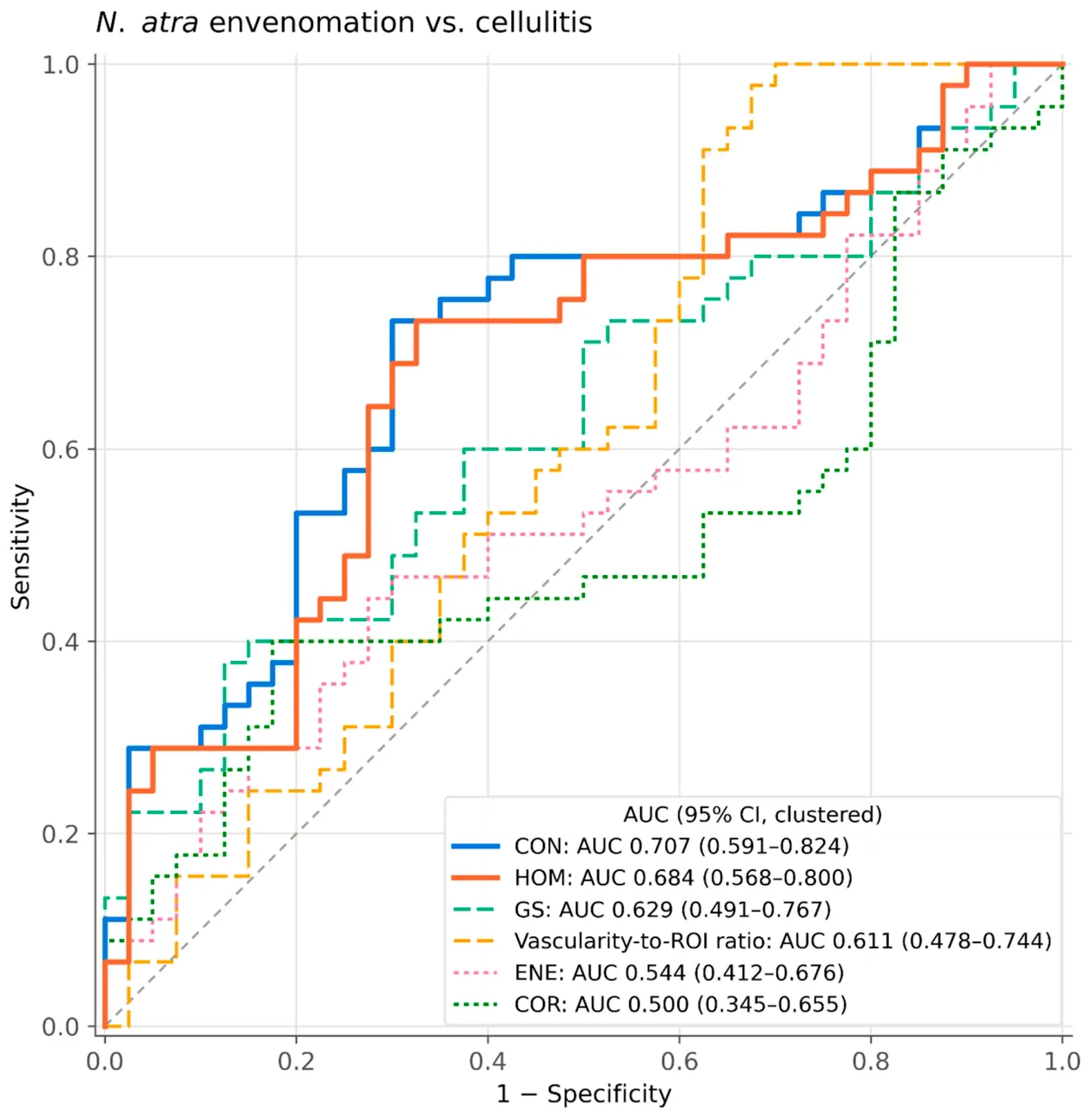
Receiver operating characteristic curves for discriminating *N. atra* envenomation from cellulitis. AUCs and 95% confidence intervals were estimated using the clustered ROC method of Obuchowski to account for multiple images from the same patient. Contrast (CON) and Homogeneity (HOM) achieved the highest discriminatory performance. The diagonal dashed line represents the line of no discrimination (AUC = 0.5).

ROC analysis for discriminating *N. atra* envenomation from cellulitis identified Contrast and Homogeneity as the features with the highest discriminatory performance among the ultrasound parameters (Figure 3). At the image level, Contrast achieved an AUC of 0.707 (95% CI, 0.591–0.824); at an optimal cutoff of ≥0.243 (higher values predicting *N. atra* envenomation), the sensitivity was 73.3%, specificity 70.0%, positive predictive value 73.3%, negative predictive value 70.0%, positive likelihood ratio 2.44, and negative likelihood ratio 0.38. Homogeneity achieved an AUC of 0.684 (95% CI, 0.568–0.800); at an optimal cutoff of ≤0.886 (lower values predicting *N. atra*), the sensitivity was 73.3%, specificity 67.5%, positive predictive value 71.7%, negative predictive value 69.2%, positive likelihood ratio 2.26, and negative likelihood ratio 0.40. Other parameters, including Correlation (AUC = 0.500) and the vascularity-to-ROI ratio (AUC = 0.611), showed lower discriminatory performance between the two disease groups. Complete diagnostic accuracy metrics for all analyzed features are presented in Table 3. In the patient-level sensitivity analysis using the first image from each patient, Contrast and Homogeneity remained significantly different between the *N. atra* envenomation and cellulitis groups (*p* = 0.022 and *p* = 0.035, respectively), with AUCs of 0.667 (95% CI, 0.525–0.808) and 0.653 (95% CI, 0.510–0.795) (Supplementary Table S1). When the image-level cutoffs were applied to the first image from each patient, Contrast yielded a sensitivity of 70.4% and a specificity of 70.0%, and Homogeneity yielded a sensitivity of 70.4% and a specificity of 67.5%. In the time-stratified analysis, time to ultrasound was not significantly correlated with Contrast (rho = 0.21, *p* = 0.299) or Homogeneity (rho = −0.23, *p* = 0.252). Images obtained within 72 h of presentation (*n* = 20) differed from the cellulitis group in the same direction, but the differences were not statistically significant (*p* = 0.224 for Contrast and *p* = 0.319 for Homogeneity). Images obtained after 72 h (*n* = 6) differed significantly from the cellulitis group (*p* = 0.005 for Contrast and *p* = 0.006 for Homogeneity) (Supplementary Table S2). Excluding the patient examined after discharge from the main analysis did not materially change the results (*p* = 0.004 for Contrast and *p* = 0.009 for Homogeneity).

## 4. Discussion

This retrospective cross-sectional study conducted at Taichung Veterans General Hospital investigated whether echotexture analysis and power Doppler vascularity can differentiate *N. atra* envenomation from cellulitis. These two conditions often present with overlapping clinical features but require fundamentally different management strategies. While most existing literature focuses on the general sonographic appearance of soft tissue infections, there is a distinct lack of research specifically utilizing quantitative ultrasound image analysis to distinguish snakebite-induced tissue injury from infectious cellulitis. The principal finding of this study is that GLCM texture features, particularly Contrast and Homogeneity, showed the greatest discriminatory ability among the parameters examined.

The preservation of significantly higher Contrast and lower Homogeneity in *N. atra* bites compared to cellulitis was the main difference between the two disease groups, and Contrast and Homogeneity remained significant after Bonferroni correction. This heterogeneously destructive pattern suggests that *N. atra* venom does not affect the tissue uniformly. Instead, it creates a complex mixture of hypoechoic necrotic pockets interspersed with relatively hyperechoic inflammatory remnants, which maintains a high level of local pixel-to-pixel intensity variation. Conversely, cellulitis was characterized by significantly lower Contrast and higher Homogeneity. Pathologically, cellulitis involves a more uniform and diffuse infiltration of inflammatory exudate throughout the soft tissue. This process effectively washes out the natural textural interfaces of the subcutaneous fat and connective tissue, leading to a smoother and more homogenized sonographic appearance. A more homogeneous texture in a swollen extremity may therefore favor bacterial cellulitis over venom-induced tissue injury, although this requires confirmation in larger cohorts. Because the ROI was confined to the subcutaneous layer, these texture differences mainly reflect changes in the subcutaneous adipose tissue. Normal subcutaneous fat consists of hypoechoic fat lobules separated by thin echogenic fibrous septa, and this lobular architecture is a major determinant of local texture. Our findings therefore suggest that venom-induced injury and bacterial infection alter the adipose tissue architecture in different ways. Future studies correlating texture features with histopathological findings may help explain these differences.

The ROC analysis identified Contrast and Homogeneity as the features with the highest discriminatory performance. Contrast achieved an area under the curve of 0.707 with an optimal cutoff of ≥0.243 for predicting *N. atra* envenomation, providing a sensitivity of 73.3% and a specificity of 70.0%. Homogeneity achieved an area under the curve of 0.684 at an optimal cutoff of ≤0.886, with a sensitivity of 73.3% and a specificity of 67.5%. These AUC values indicate moderate discriminatory performance. Contrast and Homogeneity performed better than mean grayscale intensity, which yielded an AUC of 0.629 in this study, although the confidence intervals overlapped. Because the cutoffs were derived and tested in the same dataset, the reported sensitivity and specificity are likely optimistic, and the predictive values depend on the proportion of each condition in this selected sample.

Vascularity further characterized the two conditions. Both disease groups demonstrated markedly elevated power Doppler vascularity compared with the control group, confirming a common inflammatory hyperemic response. However, the vascularity-to-ROI ratio did not significantly discriminate *N. atra* envenomation from cellulitis (*p* = 0.037, above the Bonferroni-adjusted threshold). The numerically higher vascularity observed in the cellulitis group is consistent with a robust vasodilatory response driven by bacterial infection. In contrast, *N. atra* venom may cause microvascular thrombosis and endothelial damage that partially counteract the inflammatory hyperemic response, potentially explaining why the vascular signal was not as consistently high as in cellulitis. The decision to undertake surgical intervention after *N. atra* envenomation is multifactorial and is not determined by a single imaging parameter. In a prior study from our institution, advanced age, *N. atra* bite, local ecchymosis, and bulla or blister formation were independent predictors of surgical intervention, and the ultrasound finding of absent Doppler flow was also associated with the need for surgery in univariate analysis [13]. The texture features examined here therefore reflect tissue characteristics relevant to differential diagnosis rather than a direct indicator of surgical need, and their relationship to surgical management would require integration with these established clinical predictors.

Our results align with the methodological framework established by previous research using GLCM echotexture analysis to detect structural changes in musculoskeletal tissues. A prior study achieved similar diagnostic performance, with areas under the curve ranging from 0.64 to 0.70 [10]. A notable difference is that the prior study employed a normalization ratio against an adjacent reference structure. Given that our study used raw values, future investigations should determine if a similar normalization strategy, such as computing ratios of affected to contralateral unaffected tissue, would further enhance discriminatory performance. These findings should be interpreted in terms of their intended clinical role. The method is not intended to replace history-taking, and when *N. atra* envenomation is known, treatment is directed accordingly. Rather, its value lies in supporting the evaluation of acute limb swelling when the history is ambiguous or unavailable, providing an objective texture signature that complements clinical assessment and laboratory findings. In this role, it is best regarded as a diagnostic adjunct that may help refine the decision between antivenom and antibiotic-directed management for suspected *N. atra* envenomation, rather than as a stand-alone diagnostic test. Beyond the differentiation addressed here, with larger cohorts this quantitative texture approach could be extended to distinguish envenomation by different snake species, and may also be of value in evaluating acute limb swelling of unknown cause, such as insect or bee stings.

In this exploratory time-stratified analysis, the first ultrasound images obtained more than 72 h after emergency department presentation differed significantly from those of the cellulitis group, whereas the differences in images obtained within 72 h were in the same direction but did not reach statistical significance. Given the small number of late examinations, these findings should be interpreted cautiously and do not establish a temporal increase in discriminatory performance. Whether the sonographic texture of *N. atra* envenomation changes with the progression of venom-induced tissue necrosis requires confirmation in prospective studies with serial imaging.

Secondary bacterial infection is a recognized component of local tissue injury after *N. atra* envenomation [3], and empirical antibiotics are commonly administered, so venom-induced inflammation and superimposed infection are often not clinically separable during the course of envenomation. The comparison in this study therefore reflects the clinical question of distinguishing snakebite-associated swelling, which may include superimposed infection, from non-snakebite cellulitis. If superimposed infection were the main determinant of the texture features, images obtained later in the clinical course, when infection is more likely, would be expected to resemble cellulitis more closely. In the exploratory time-stratified analysis, later images did not appear more similar to cellulitis, which does not suggest that superimposed infection was the main explanation for the texture differences, although this observation is based on a small number of late examinations.

## 5. Limitations

Several limitations must be acknowledged. First, this was a retrospective single-center study conducted at Taichung Veterans General Hospital with a sample size of 115 images from 84 patients. This relatively small cohort may lead to low statistical power, potentially limiting the precision of our diagnostic thresholds and the generalizability of the findings. Second, while the identification of the envenoming species could be viewed as a presumed identity in some clinical records, we relied on visual evidence (specimens or photographs) rather than molecular confirmation; however, the monotypic distribution of *N. atra* in Taiwan reduces the risk of misidentification within the genus *Naja*. Third, no normalization strategy was applied to account for potential inter-patient variability in tissue echogenicity, and differences in gain or preset selection can affect grayscale and contrast through changes in dynamic range, potentially influencing texture features; however, the use of a single ultrasound system and a standardized acquisition protocol throughout the study is expected to have minimized this variability. Fourth, because secondary bacterial infection can complicate *N. atra* envenomation, the envenomation group may include cases with superimposed cellulitis-like changes. We consider this intrinsic to the clinical condition under study rather than a misclassification, because the comparison was designed to separate snakebite-associated swelling from cellulitis of non-snakebite origin. The relative contributions of venom-induced tissue injury and superimposed infection to the texture features could not be separated, and prospective studies with microbiological assessment would help clarify this issue. Fifth, standardized serial images were not available, and the exact interval from the bite or symptom onset to the ultrasound examination could not be consistently determined. Because the sonographic features of envenomation and cellulitis may evolve over time, prospective time-series studies would help characterize how these texture features change over the clinical course. In addition, the number of examinations obtained more than 72 h after presentation was small, and comparable timing data were not available for the cellulitis group. Sixth, the optimal cutoffs were derived and evaluated in the same dataset without internal or external validation, and the predictive values reflect the case mix of this retrospective sample. These accuracy estimates should therefore be regarded as exploratory. Seventh, the ROIs were delineated by the same rheumatologist who performed the examinations. Although ROI delineation was performed retrospectively on de-identified images after a substantial interval, complete blinding to the clinical diagnosis cannot be ensured. Finally, although the distribution of anatomical sites was comparable between the two disease groups, site-related differences in baseline texture and Doppler signal cannot be fully excluded.

Despite these limitations, this study provides proof-of-concept that quantitative echotexture analysis can detect pathological changes in acute soft tissue conditions and suggests that it may help differentiate *N. atra* envenomation from cellulitis. The Contrast and Homogeneity features, at optimal cutoffs achieving approximately 70% sensitivity and specificity, represent a starting point. Future prospective studies should incorporate larger sample sizes, contralateral tissue normalization, multi-angle GLCM analysis, and integration of echotexture features with clinical variables (bite history, laboratory markers) into multivariate diagnostic models, and correlation of quantitative grayscale and texture features with the severity of limb swelling and clinical outcomes. The potential for integration with artificial intelligence-assisted POCUS workflows could further enhance the clinical applicability of this approach in settings where *N. atra* envenomation and cellulitis coexist as diagnostic challenges.

## 6. Conclusions

In conclusion, this study suggests that quantitative ultrasound texture analysis using gray-level co-occurrence matrix features may help differentiate *N. atra* envenomation from cellulitis. While both conditions exhibit significantly increased vascularity and altered texture compared with the control group, the distinct patterns of Contrast and Homogeneity were the features that best discriminated between these two pathological states. *N. atra* bites are characterized by a heterogeneously destructive texture with higher Contrast and lower Homogeneity than cellulitis, which may reflect the complex tissue damage induced by venom. These proof-of-concept findings support further investigation of quantitative echotexture analysis as a potential bedside diagnostic aid for acute limb swelling in snake-endemic regions, with prospective validation in larger cohorts required to establish clinical utility.

## Figures and Tables

**Table 1:** Demographic and clinical characteristics of the *N. atra* envenomation and cellulitis groups.

	*N. atra* Envenomation (*n* = 27)	Cellulitis (*n* = 40)	*p* Value
Male, *n* (%)	17 (63.0)	24 (60.0)	0.807
Female, *n* (%)	10 (37.0)	16 (40.0)	
Age, years	55.9 ± 16.5	61.7 ± 18.1	0.150
WBC, /µL	8300 (6415–9330)	7340 (6015–11,045)	0.764
Neutrophils, %	76.9 (62.0–85.1)	69.4 (64.0–80.4)	0.657
Lymphocytes, %	18.0 (9.0–30.5)	21.1 (12.1–25.9)	0.932
CRP, mg/dL	0.15 (0.08–1.14)	1.42 (0.30–3.34)	0.009

Age is presented as mean ± standard deviation and laboratory parameters as median (interquartile range). *p* values were derived from the Mann–Whitney U test for continuous variables and the chi-square test for sex. Data were available for WBC, neutrophils, and lymphocytes in 39 cellulitis patients, and for CRP in 34 cellulitis patients. CRP values below the detection limit in the envenomation group were assigned the detection-limit value. WBC, white blood cell count; CRP, C-reactive protein.

**Table 2:** Quantitative Ultrasound Features by Group.

Parameters	*N. atra* Envenomation (45 Images, 27 Patients)	Cellulitis (40 Images, 40 Patients)	Control (30 Images, 17 Patients)	Overall *p*	*p* (*N. atra* vs. Control)	*p* (Cellulitis vs. Control)	*p* (*N. atra* vs. Cellulitis)
GS value	88.64 ± 22.39 86.73 (71.36–101.26)	98.99 ± 22.85 95.68 (84.27–109.52)	106.59 ± 28.53 112.01 (88.60–127.26)	0.139	0.048	0.213	0.226
CON	0.273 ± 0.068 0.258 (0.239–0.309)	0.230 ± 0.040 0.230 (0.210–0.251)	0.274 ± 0.065 0.253 (0.227–0.320)	<0.001	0.816	0.009	0.001
COR	0.911 ± 0.044 0.926 (0.882–0.944)	0.917 ± 0.028 0.916 (0.903–0.935)	0.886 ± 0.036 0.890 (0.853–0.918)	<0.001	0.014	<0.001	0.270
ENE	0.156 ± 0.041 0.153 (0.122–0.190)	0.167 ± 0.046 0.162 (0.134–0.183)	0.177 ± 0.045 0.176 (0.142–0.208)	0.247	0.100	0.329	0.335
HOM	0.878 ± 0.020 0.880 (0.867–0.888)	0.890 ± 0.017 0.889 (0.880–0.896)	0.874 ± 0.025 0.883 (0.856–0.890)	0.005	0.428	0.016	0.004
Vascularity-to-ROI ratio (%)	2.69 ± 2.22 2.08 (0.92–4.04)	6.87 ± 8.41 3.09 (1.14–10.19)	0.06 ± 0.30 0.00 (0.00–0.00)	<0.001	<0.001	<0.001	0.037

Values are presented as mean ± SD (upper line) and median (IQR) (lower line) at the image level. *p* values were derived from generalized estimating equations with an exchangeable working correlation structure to account for multiple images obtained from the same patient. The overall *p* value is from a Wald test of group. The vascularity-to-ROI ratio was expressed as the percentage of color pixels within the ROI and was log-transformed (log[x + 1]) for modeling. Unadjusted *p* values are shown. For pairwise comparisons, statistical significance was assessed using a Bonferroni-adjusted threshold of *p* < 0.017. GS, grayscale. CON, contrast. COR, correlation. ENE, energy. HOM, homogeneity. ROI, region of interest.

**Table 3:** Diagnostic Accuracy of Echotexture Features for Discriminating *N. atra* Envenomation from Cellulitis.

Feature	AUC (95% CI)	Cutoff	ACC %	SEN %	SPE %	PPV %	NPV %	LR+	LR−
GS	0.629 (0.491–0.767)	≤76.86	61.18	37.78	87.50	77.27	55.56	3.02	0.71
CON	0.707 (0.591–0.824)	≥0.243	71.76	73.33	70.00	73.33	70.00	2.44	0.38
COR	0.500 (0.345–0.655)	≤0.892	60.00	40.00	82.50	72.00	55.00	2.29	0.73
ENE	0.544 (0.412–0.676)	≤0.142	57.65	44.44	72.50	64.52	53.70	1.62	0.77
HOM	0.684 (0.568–0.800)	≤0.886	70.59	73.33	67.50	71.74	69.23	2.26	0.40
Vascularity-to-ROI ratio (%)	0.611 (0.478–0.744)	≤7.01	67.06	97.78	32.50	61.97	92.86	1.45	0.07

Diagnostic accuracy was calculated at the image level (45 *N. atra* envenomation and 40 cellulitis images). The 95% CIs of the AUC were estimated using the nonparametric method for clustered ROC data described by Obuchowski, to account for multiple images obtained from the same patient. Optimal cutoffs were determined by the Youden index. ≥ indicates that values at or above the cutoff predict *N. atra* envenomation, and ≤ indicates that values at or below the cutoff predict *N. atra* envenomation. Because the cutoffs were derived and evaluated in the same dataset, accuracy estimates are likely optimistic, and predictive values reflect the proportion of each condition in this sample. The vascularity-to-ROI ratio is expressed as the percentage of color pixels within the ROI. AUC, area under the receiver operating characteristic curve. CI, confidence interval. ACC, accuracy. SEN, sensitivity. SPE, specificity. PPV, positive predictive value. NPV, negative predictive value. LR+, positive likelihood ratio. LR−, negative likelihood ratio. ROI, region of interest.

## Data Availability

The data that support the findings of this study are available from the corresponding author upon reasonable request.

## Supplementary Materials

The following supporting information can be downloaded at: https://www.mdpi.com/article/10.3390/diagnostics16213594/s1, Figure S1: Study flow diagram; Table S1: Patient-level sensitivity analysis using the first ultrasound image from each patient; Table S2: Time-stratified comparison of contrast and homogeneity between the *N. atra* envenomation and cellulitis groups.
