# Supplementary material for: Ultrasound Echotexture Analysis as a Diagnostic Adjunct for Naja atra Envenomation Versus Cellulitis in Acute Limb Swelling
Source: Diagnostics (Basel). 2026 Oct 30;16(21):3594. doi: 10.3390/diagnostics16213594 (PMC13762326; doi:10.3390/diagnostics16213594)
Supplement: Supplementary file 1 [file diagnostics-16-03594-s001.zip › diagnostics-4549878-supplementary.pdf]

Supplementary Materials

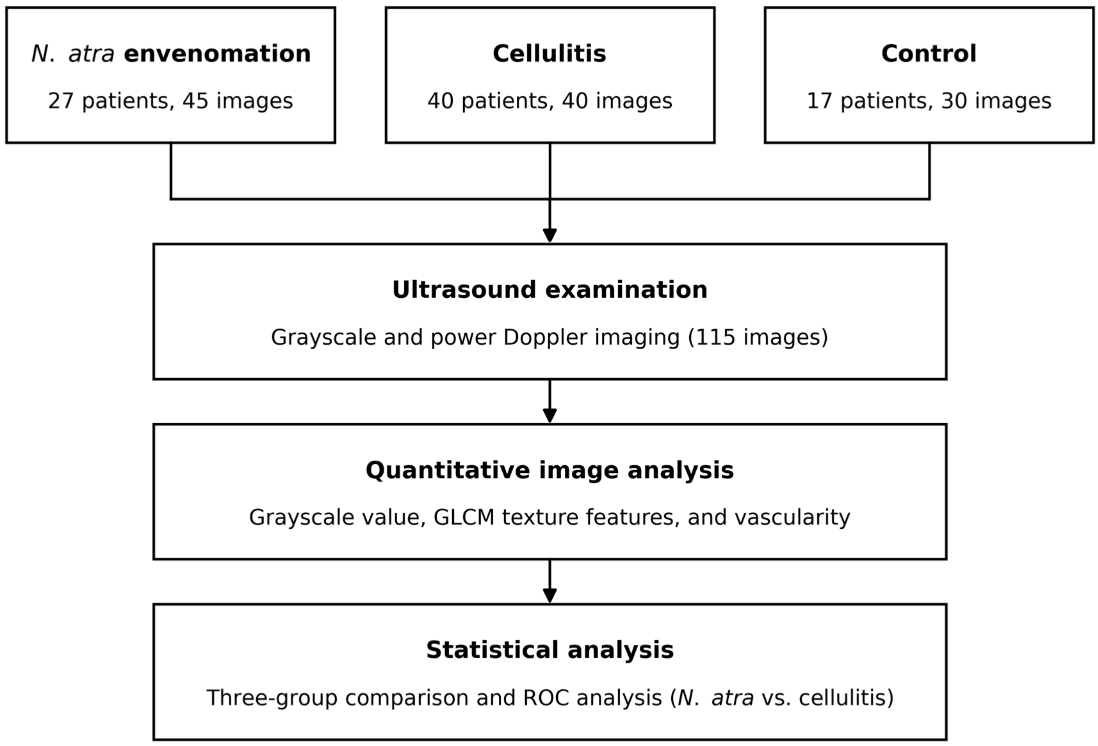

Figure S1. Study flow diagram.

GLCM, gray-level co-occurrence matrix. ROC, receiver operating characteristic.

Table S1. Patient-level sensitivity analysis using the first ultrasound image from each patient.

Parameter	*N. atra* envenomation (n = 27)	Cellulitis (n = 40)	p value	AUC (95% CI)
GS value	94.07 (77.96–113.16)	95.68 (84.27–109.52)	0.735	0.525 (0.376–0.674)
CON	0.260 (0.219–0.308)	0.230 (0.210–0.251)	0.022	0.667 (0.525–0.808)
COR	0.910 (0.882–0.942)	0.916 (0.903–0.935)	0.462	0.554 (0.401–0.707)
ENE	0.166 (0.122–0.191)	0.162 (0.134–0.183)	0.843	0.515 (0.367–0.662)
HOM	0.880 (0.868–0.895)	0.889 (0.880–0.896)	0.035	0.653 (0.510–0.795)
Vascularity-to-ROI ratio (%)	3.33 (0.69–4.82)	3.09 (1.14–10.19)	0.247	0.584 (0.445–0.723)

Values are presented as median (IQR). Unadjusted p values were derived from the Mann-Whitney U test. The 95% CIs of the AUC were estimated using the DeLong method. Applying the cutoffs in Table 3 to this patient-level dataset, CON ≥ 0.243 yielded a sensitivity of 70.4% and a specificity of 70.0%, and HOM ≤ 0.886 yielded a sensitivity of 70.4% and a specificity of 67.5%. The vascularity-to-ROI ratio is expressed as the percentage of color pixels within the region of interest. AUC, area under the receiver operating characteristic curve. CI, confidence interval. GS, grayscale. CON, contrast. COR, correlation. ENE, energy. HOM, homogeneity. IQR, interquartile range.

Table S2. Time-stratified comparison of contrast and homogeneity between the *N. atra* envenomation and cellulitis groups.

Group	n	CON, median (IQR)	p value	AUC	HOM, median (IQR)	p value	AUC
Cellulitis	40	0.230 (0.210–0.251)			0.889 (0.880–0.896)		
N. atra, ≤ 72 h	20	0.248 (0.216–0.262)	0.224	0.598	0.881 (0.877–0.897)	0.319	0.580
N. atra, > 72 h	6	0.316 (0.309–0.329)	0.005	0.842	0.858 (0.854–0.866)	0.006	0.838

The first ultrasound image from each *N. atra* envenomation patient was stratified by the time from emergency department presentation to examination. One patient examined at an outpatient visit after discharge was excluded. In this exploratory analysis, unadjusted p values were derived from the Mann-Whitney U test comparing each stratum with the cellulitis group, and AUCs refer to discrimination from the cellulitis group. Across the *N. atra* envenomation group, time to ultrasound was not significantly correlated with CON (Spearman rho = 0.21, p = 0.299) or HOM (rho = -0.23, p = 0.252). AUC, area under the receiver operating characteristic curve. CON, contrast. HOM, homogeneity. IQR, interquartile range.
